# Tips to Ensure Optimal Ring Apposition of the Ovation Stent Graft in Challenging Necks of Abdominal Aortic Aneurysms

**DOI:** 10.1055/s-0039-1688434

**Published:** 2019-09-17

**Authors:** Efstratios Georgakarakos, Andreas Koutsoumpelis, Kalliopi-Maria Tasopoulou, George S. Georgiadis

**Affiliations:** 1 Department of Vascular Surgery, University Hospital of Alexandroupolis, “Democritus” University of Thrace, Alexandroupolis, Greece

**Keywords:** EVAR, abdominal aortic aneurysm, Ovation, stent graft, aorta, technical maneuvers

## Abstract

The Ovation stent graft has been recently introduced for endovascular repair of abdominal aortic aneurysms. Its sealing mechanism is based on a pair of polymer-filled inflatable rings. Based on our experience, we describe useful tips to optimize the use of Ovation in thrombosed or severely angulated necks.

## Introduction

The Ovation Abdominal Stent Graft system (Endologix, Irvine, CA) is a trimodular endoprosthesis introduced for endovascular repair of abdominal aortic aneurysms (AAAs). 1 This endograft dissociates the stages of sealing and fixation with the former being accomplished infrarenally by a pair of polymer-filled inflatable rings and the fixation achieved via a 35-mm-long suprarenal stent. 2 A network of compliant, inflatable sealing O-rings are filled with a low-viscosity radiopaque polymer during the endograft's deployment to the point of accommodating tightly to the luminal surface of the AAA neck, thus providing an effective, gasket-like sealing effect. 2 The endograft is accommodated in a low profile 14 Fr delivery system. In this article, we describe our technical approach to the management of heavily thrombosed or severely angulated necks with this particular endograft.

## Techniques

### Patient 1

**Fig. 1:**
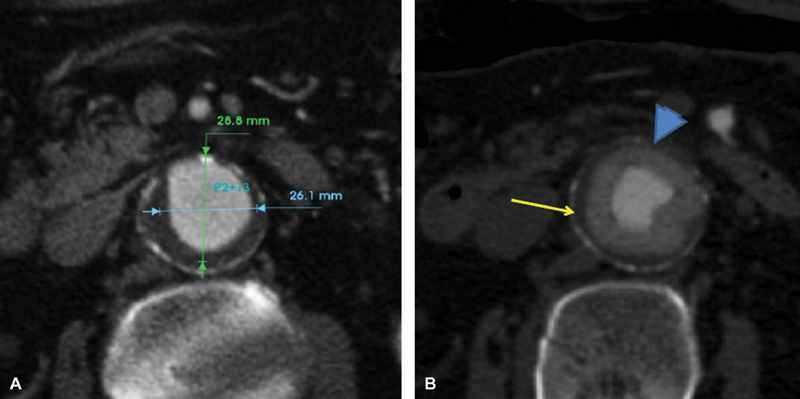
( **A** ) Case 1 showing an aneurysm with excessive thrombus at the infrarenal sealing level. ( **B** ) The inflated polymer-filled sealing rings ( *arrowhead* ) after distension with molding balloon. Note the compressed neck thrombus ( *arrow* ).

A 78-year-old male patient underwent endovascular aortic aneurysm repair. Τhere was a significant amount of eccentric thrombus ( Fig. 1A ) in the infrarenal neck, rendering the effective sealing with a nitinol-based endograft dubious. Therefore, a 34-mm Ovation (Endologix) device was used to achieve effective sealing with the inflatable polymer-filled rings. Immediately after completion of polymer injection, gradual insufflation of the rings was achieved with a molding balloon ( Fig. 1B ), leading to a notable compression of the thrombus against the neck surface (arrow), optimal apposition of the rings (arrowhead), and ideal sealing.

### Patient 2

**Fig. 2:**
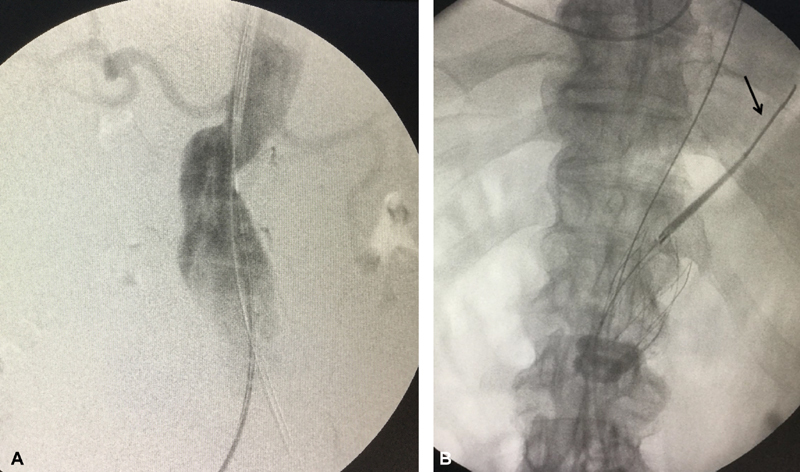
( **A** ) Intraoperative angiography of case 2 showing significant angulation of the infarenal neck and insertion of super-stiff guidewires bilaterally. ( **B** ) Polymer filling of the sealing rings of Ovation. Note that the super-stiff guidewire has been withdrawn until its soft cephalad tip approaches the nose cone of the delivery system.

An 82-year-old male patient was treated with Ovation for an AAA of 55 mm. The infarenal neck had a 23-mm diameter at the sealing level and a marked angulation of 60 degrees ( Fig. 2A ). Insertion of super-stiff guidewires can cause straightening of the infrarenal neck, altering its angulation on consequent polymer infusion and sealing of the inflatable rings. However, the neck geometry may resume its original shape once the guidewires are removed. Thus, it is our suggestion to withdraw the super-stiff guidewire supporting the endograft until the soft cephalad tip approaches the nose cone of the delivery system ( Fig. 2B ). This way, the infrarenal angle approaches its original shape and the sealing of the rings is achieved, firmly reducing any postoperative tension and/or strain due to neck remodeling.

## Discussion

The Ovation stent graft expands the AAA eligibility for endovascular aneurysm repair by 10%, overcoming classic limitations of the infrarenal neck such as conical shape, excessive thrombus, significant calcification, and length of ≤ 10 to 15 mm. Although the postoperative adverse event rate has been documented to be quite low, treating AAA with challenging necks at the verge or outside the instructions-for-use has been associated with central Type IA endoleaks. 3 4 5

We presented two cases of challenging neck instances, where we tend to modify our deployment of sealing technique. In heavily eccentrically thrombosed necks, there are questionable issues regarding the applicability and degree of oversizing of any given endograft. The thrombus layers are not of equal density or mechanical properties. 6 7 Such issues tend to render questions about the use of nitinol-based endografts, while the low outward force of the gasket-like effect of the Ovation's polymer-filled rings makes the latter suitable for such cases. In our practice, we tend to insufflate the rings gradually with a molding balloon to compress the luminal thrombus' “loose” layers, leaving behind only the outer dense layers where the solidified O-rings of the endograft will be positioned. The central nitinol narrow zone of Ovation (first zone of sealing) is not affected and can, therefore, prevent any cephalad dislodgment of thrombus to the renal arteries. Furthermore, this maneuver limits the inward protrusion of the inflated rings, which can induce flow lumen stenosis, especially in the presence of significant amount of thrombus. 8

The second case presents marked angulation of the infrarenal neck. Since angulation has been recognized as determinant of Type IA endoleak, we pay attention to inflation of the rings as close as possible to the tension-free postoperative neck geometry, to avoid immediate change of orientation of the inflated rings. 9 Therefore, in severely angulated necks we prefer withdrawing the super-stiff guidewire so that the rings are oriented so as to inflate somewhat obliquely to the actual infrarenal lumen centerline.

In our experience, the technical tips described above can be expected to facilitate the optimal sealing of Ovation in challenging necks with marked infrarenal thrombus or significant angulation.
